# Allostatic Load and Biological Aging Indicators in the MIDUS National Survey

**DOI:** 10.1093/geroni/igaa057.1694

**Published:** 2020-12-16

**Authors:** Waylon Hastings, Idan Shalev, David Almeida

**Affiliations:** Pennsylvania State University, University Park, Pennsylvania, United States

## Abstract

Indices quantifying allostatic load (AL) and biological aging (BA) have received widespread use in epidemiological and health science literature. However, little attention has been paid to the conceptual and quantitative overlap between these indicators. By reviewing literature utilizing measures of AL and BA, we highlight differences with respect to biological markers employed and approach toward scale construction. Further, we outline opportunities where AL indices might be improved by adopting analytical features of BA measures. We demonstrate the utility of this approach using data from The MIDUS National Survey, constructing three indices of allostatic load: one standard approach modeled after Gruenewald et al, 2012, and two alternative formulations informed by BA procedures. The performance of AL indices are juxtaposed against two commonly employed indices of biological aging: Klemera-Doubal Method Biological Age and Homeostatic Dysregulation. All measures were significantly associated with chronological age. Alternative AL formulations were more strongly associated with biological aging measures than with the standard approach. MIDUS participants with increased allostatic load and older biological ages performed worse on tests of physical, cognitive, perceptual, and subjective functioning. Further, MIDUS participants with history of childhood-trauma and mental-health problems were measured as having increased AL and BA. Alternative AL formulations tended to have effect-sizes equivalent to or larger than those observed for BA measures. In conclusion, indices of allostatic load and biological age approximate similar processes when constructed with comparable biomarkers and rigor, in line with their conceptual overlap as proxies of cumulative wear and tear.

